# Editorial: Reviews in gastroenterology

**DOI:** 10.3389/fmed.2023.1200204

**Published:** 2023-04-28

**Authors:** Tian Lan, Brooke R. Druliner, Enis Kostallari, Huan Tong

**Affiliations:** ^1^Laboratory of Gastroenterology and Hepatology, West China Hospital, Sichuan University, Chengdu, China; ^2^Department of Gastroenterology and Hepatology, West China Hospital, Sichuan University, Chengdu, China; ^3^Division of Gastroenterology and Hepatology, Mayo Clinic, Rochester, MN, United States

**Keywords:** gastroenterology, hepatology, pancreatobiliary disease, gastrointestinal tumor, reviews, non-alcoholic fatty liver disease, acute-on-chronic liver failure

## Introduction

Gastroenterology is a rapidly evolving discipline which covers diseases of the digestive tract (esophagus, stomach, and intestine) and digestive glands (liver, biliary tree, and pancreas). This Research Topic presents eight reviews which focus on the following specific conditions and diseases: gastroesophageal reflux disease, implications following cholecystectomy, locally advanced right colon cancer, appendiceal cancer, enteral nutrition, lumen-apposing metal stent, non-alcoholic fatty liver disease, and acute-on-chronic liver failure. Each of these complications, conditions and diseases account for an overwhelming disease burden in the fields of gastroenterology and hepatology.

## Summary of the reviews

The standard treatments for gastroesophageal reflux disease (GERD) are lifestyle adjustment and proton pump inhibitors. The agonist of the gamma-aminobutyric acid receptor, baclofen reduces reflux by inhibiting lower esophageal sphincter relaxation, and is used as an alternative management for GERD (1). However, the efficacy and side effects of baclofen in GERD treatment still need investigations. Arabpour et al. systemically reviewed 26 clinical trials and highlighted that baclofen benefited four populations with GERD as below: adults, children, patients with gastroesophageal reflux-induced chronic cough, and hiatal hernia patients. Baclofen ameliorates reflux symptoms, as well as improves pH-monitoring and manometry results. It should be noted that most of the published clinical studies on baclofen treatment in GERD had small sample sizes, and approximately one third of them were not randomized clinical trials. Although baclofen seems to be a promising medication and diversifies the treatment of GERD, the use of baclofen in the treatment of GERD needs further investigation.

Cholecystectomy can disturb bile flow and bile acid circulation, thus dysregulating gut microbiota (2). Growing evidence has linked these changes after cholecystectomy to the development of colorectal cancer (CRC) (3, 4). Jiang et al. discussed how cholecystectomy results in altered bile acid homeostasis and gut microbiota. In addition, they highlight the mechanisms involved in these changes, which pose a higher risk of CRC compared to non-cholecystectomized patients. The authors also summarized the potential tumorigenic effect of secondary bile acids on CRC. The potential of bile acid treatment for CRC was also discussed. As there are a considerable number of patients receiving cholecystectomy worldwide, it alerts that these patients have a higher risk for CRC, and it is meaningful to enhance CRC screening in these populations.

Locally advanced right colon cancer (LARCC) invades neighboring organs such as the pancreas and the duodenum, which challenges surgical interventions (5). En bloc resection is considered a suitable surgical method for this situation but strong evidence from clinical trials is lacking (6). To identify the effect of en bloc resection in LARCC cases with neighboring organ invasion, the data of 117 patients showed that en bloc resection plus right hemicolectomy was superior to pancreaticoduodenectomy with respect to survival of LARCC (Ri et al.). Although more data are needed to validate this conclusion, en bloc resection plus right hemicolectomy might be a better surgical procedure for LARCC.

Appendiceal cancer is a rare gastrointestinal tumor with limited knowledge on its risk stratification, which hinders treatment strategies for this disease. To this end, Liu et al. collected and analyzed the clinical data from a cohort with a large number of patients with primary appendiceal cancer from the Surveillance, Epidemiology, and End Results database. Age, pathological stage, surgery, number of lymph nodes removed, T stage, N stage, M stage, and CEA are the independent prognosis factors for appendiceal cancer. A monogram survival prediction model was established and validated to predict 1, 3, and 5-year overall survival for patients with appendiceal cancer and stratify the risk to give the individualized treatment.

Enteral nutrition (EN) consists of the delivery of nutrients via tubes and is suggested as the first-line treatment to induce remission in pediatric inflammatory bowel disease (IBD) (7). However, the application of specific EN, such as exclusive EN, has not reached a consensus. Luo et al. reviewed 12 questionnaire survey studies regarding exclusive EN application in pediatric IBD, and summarized current opinions on EN methods, including treatment course, formula, and food reintroduction, which might help to standardize the use of EN in children IBD.

The lumen-apposing metal stent (LAMS) is a large-channel metal stent used in endoscopic ultrasonic-guided intervention and has been applied recently for pancreatic fluid collection drainage (8). However, this method has suffered from several drawbacks, such as complicated and difficult procedures. Yi et al. introduced a recently developed endoscopic approach combining LAMS and electro-cautery cyctotome, namely electro-cautery LAMS (EC-LAMS). EC-LAMS shows several advantages compared to conventional LAMS and double pigtail plastic stents, including easy deployment, lower cost, shorter processing time, broader indications, and lower risk of complications such as bleeding. In general, EC-LAMS seems to be a novel and promising method for stent placing.

Non-alcoholic fatty liver disease (NAFLD) has become the most common cause of chronic liver disease with increasing disease burden (9, 10). Grip strength (GS) is an index of muscle strength and is associated with metabolic function (11). As NAFLD patients are often accompanied by metabolic disorders, GS might be considered as an indicator of NAFLD. Han et al. systemically reviewed 10 cross-section studies to reveal the relationship between GS and NAFLD. They demonstrated that people with low GS have higher risk of NAFLD, while NAFLD patients are associated to lower GS than healthy population. However, these cross-section studies were only conducted in China and Korea, and it is still unclear whether these conclusions can be applied in other populations.

Acute-on-chronic liver failure (ACLF) is caused by acute decompensation based on chronic liver diseases (12). Laboratory studies are trying to clarify the pathogenesis of ACLF and discover potential therapeutic targets. However, one of the major obstacles is the lack of suitable ACLF animal models. To this end, Zhai et al. compared the characteristics of current ACLF mouse models. Moreover, they established an ACLF mouse model by sequential injection of carbon tetrachloride (CCl_4_) for 8 weeks, followed by a double dosage of CCl_4_ for 72 hours, and *Klebsiella pneumoniae* administration. This model recapitulates the usual clinical course of human ACLF and represents a potential animal model for future ACLF studies.

## Conclusion

The reviews of this special issue summarize the recent advances in the field of gastroenterology and hepatology. These reviews encompass a wide range of diseases spanning the gastrointestinal tract and the liver. We are optimistic that the knowledge gaps identified by these reviews are the first step toward delivering better care for patients.

## Author contributions

All authors drafted the manuscript. EK and HT revised the manuscript. All authors contributed to the article and approved the submitted version.
